# The antiepileptic effect of Gastrodiae Rhizoma through modulating overexpression of mTOR and attenuating astrogliosis in pilocarpine mice model

**DOI:** 10.1002/epi4.12372

**Published:** 2019-12-13

**Authors:** Ka Lai Yip, Chi Man Koon, Zi Yi Chen, Ping Chook, Ping Chung Leung, Steven Schachter, Wai Hong Leung, Chung Tong Mok, Howan Leung

**Affiliations:** ^1^ Department of Medicine and Therapeutics The Chinese University of Hong Kong New Territories Hong Kong; ^2^ Institute of Chinese Medicine The Chinese University of Hong Kong New Territories Hong Kong; ^3^ State Key Laboratory of Research on Bioactivities and Clinical Applications of Medicinal Plants The Chinese University of Hong Kong New Territories Hong Kong; ^4^ Department of Neurology The First Affiliated Hospital Sun Yat‐sen University Guangzhou China; ^5^ Beth Israel Deaconess Medical Center Harvard Medical School Boston MA USA; ^6^ Center for Integration of Medicine and Innovative Technology Massachusetts General Hospital Boston MA USA

**Keywords:** antiepileptic, astrogliosis, Gastrodiae Rhizoma, mTOR, neuronal atrophy, SEMA3F

## Abstract

**Objective:**

To investigate the effect of water extract of Gastrodiae Rhizoma (GR) on the development of acquired temporal lobe epilepsy (TLE) and on regulating the expression of the mammalian target of rapamycin (mTOR) and semaphorin 3F (SEMA3F).

**Methods:**

A pilocarpine‐induced status epilepticus (SE) model was adopted to precipitate injury in the limbic systems. GR and carbamazepine (CBZ) treatments were given to mice for 14 days prior to SE induction to demonstrate the antiepileptic effects and continued for 5 more days to illustrate the effects on histologic studies.

**Results:**

Our results consolidated that GR treatment (92.1 minutes) could delay the SE onset in comparison with the control group (61.5 minutes, *P* = .041). Fewer mice had reached SE with GR treatment (41.7%) when compared with the control group (83.3%, *P* = .044). GR treatment (2.1 hours/mouse) could suppress the number of acute seizures in post‐SE survival mice when compared with the control group (4.5 hours/mouse, *P* < .001). The effects of GR treatment were elucidated with the mechanism of actions. GR treatment reduced the overexpression of mTOR (0.27 vs 0.67 AU/mg protein, *P* = .047). GR treatment increased the underexpression of SEMA3F (0.51 vs 0.16 µg/mg protein, *P* = .034). In the histochemical study of microtubule‐associated protein 2 (MAP2) staining, our results showed that GR prevented neuronal loss in the GR treatment group (64.8% positively stained pixel area) as compared with the control group (59%, *P* = .014) in the hippocampus. In glial fibrillary acidic protein (GFAP) staining, the severity of astrogliosis was mitigated by the GR treatment (4.1% positively stained pixel area) when compared to the control group (5.6%, *P* = .047) in the hippocampus.

**Significance:**

These results provide preclinical evidence to support the use of GR, which could suppress acute seizures and relieve pathological changes in pilocarpine‐induced TLE mice. We demonstrated that the antiepileptic effects of GR could be accompanied by mTOR reduction and astrogliosis attenuation.

**Key Points**

- Water extract of Gastrodiae Rhizoma (GR) delays the onset of pilocarpine‐induced status epilepticus and could suppress the acute seizures in temporal lobe epilepsy (TLE) mice
- The treatment of GR alleviates neuronal loss and mitigates the severity of astrogliosis in the hippocampal region
- The mechanism involves the reduction of overexpression of mTOR and the upregulation of underexpression of SEMA3F

## 1 INTRODUCTION

Gastrodiae Rhizoma (GR, Tianma) is the rhizome of *Gastrodia elata* Blume and traditional Chinese medicine (TCM), which is documented in the Chinese Pharmacopoeia. In particular, GR has been implicated in the treatment of a variety of neurological conditions, with seizure being the most notable one.^1^ To date, approximately 81 constituents of GR have been isolated with different solvents and extraction methods; the major bioactive compound is gastrodin in terms of therapeutic use.^2^

In a preclinical evaluation of epilepsy treatment, past literature showed that the extracts of GR and its constituents had antioxidant and scavenging free radical activities.^3^, ^4^ Studies from Hsieh et al indicated anticonvulsive effects of GR—showing that extract of GR could reduce convulsive syndrome in a pretreatment manner of kainic acid‐induced epileptic rat model.^5^, ^6^, ^7^ Subsequent studies confirmed that GR might reduce the severity of status epilepticus (SE), as well as seizure frequency, and could protect the neuronal damage against kainic acid in mouse hippocampus.^8^, ^9^ Two mechanisms have been proposed for its effects: Hsieh et al suggested a signaling pathway that GR modulated mitogen‐activated protein kinase (MAPK) pathway to regulate activator protein 1 (AP‐1) expression after SE induction, and Shao et al reported that gastrodin acted on downregulating the expression of Nav1.6 channel protein in pretreatment manner of pilocarpine‐induced temporal lobe epilepsy (TLE) in rats.^9^, ^10^ Moreover, recent literature documented that gastrodin ameliorated pentylenetetrazole (PTZ)‐induced seizure with an improvement of electroencephalographic outcomes in mice.^11^ These results support the potential therapeutic effects of GR in epilepsy.

Despite these encouraging evidences, the mechanism of GR has not been mapped precisely and remained unclear. This may be a missing link from benches to bedside, thus leading to rare clinical practices. We aimed to investigate the expression of proteins that might relate to epilepsy—mammalian target of rapamycin (mTOR) and semaphorin 3F (SEMA3F), respectively—for establishing the mechanism and explaining the antiepileptic effect of GR. The expression level of phosphorylated mTOR (p‐mTOR) may reveal a mechanistic pathway in epilepsy formation and its severity. SEMA3F governs the axonal growth and synaptic formation, which involves in early axonal sprouting, as well as astrogliosis. A wide range of pretreatment duration was given for investigating the effects of GR in TLE model, from a dose of 30 minutes ahead of epilepsy induction to as much as 9 days, and only one study extended the pretreatment period to 14 days.^7^, ^8^, ^9^ Our choice of a 14 day pretreatment scheme may be seen as a long treatment period for the model of TLE. We adopted the pretreatment period of 14 days that may help to determine the tolerability and toxicity of GR by assessing the survival rate in the acute approach of toxicity test.^12^

For this study, we chose water extraction, which would minimize the safety concern to mice and the absorption of GR. In particular, water extraction of GR has been limitedly reported on epilepsy. The effects of GR extract were compared with carbamazepine, a conventional antiepileptic drug (AED) by a widely used TLE model—pilocarpine‐induced SE, which was first described by Turski et al.^13^ This is a convincing model for drug screening with clinical features resembling.^14^

## 2 MATERIALS AND METHODS

### 2.1 Herbal material and aqueous extraction

The experimental batch of GR (voucher specimen number: 20113351) was grown in Hubei province and obtained from Zhixin Medicine Health Co. Ltd. in Guangdong, China. The herbal material was authenticated in accordance with Chinese Pharmacopoeia (2015) by layer chromatography. Ten kilograms GR was washed by soaking in 10‐fold distilled water (w/v) for one hour. It was extracted in 1:10 distilled water (w/v) at 100°C for two rounds. Each round of extraction lasted for 1 hour. The combined residue was filtered. The extract of GR was concentrated in reduced pressure at 60°C and lyophilized into dry powder. The extraction yield was 43.1%.

### 2.2 Animals

Experimental procedures were approved by the Animal Experimentation Ethics Committee of the Chinese University of Hong Kong (CUHK). Adult male C57BL/6 mice, aged between 8 and 10 weeks (weighing 22‐30 g), were supplied by The Laboratory of Animal Services Centre (LASEC) of CUHK. Three mice were housed in a cage and given food and water ad libitum. The environment was maintained in 12‐hour light‐dark cycles. The mice were randomly divided into 4 groups: naïve, control, CBZ, and GR for different treatments. No additional mouse was used for compensation of the death due to any cause.

### 2.3 Dose determination and administration

The dose of GR for mice was calculated according to Chinese Pharmacopoeia,^15^ which equaled to 883.56 mg/kg daily. The GR group received 883.56 mg/kg GR extract daily, and the CBZ group received 200 mg/kg CBZ daily. The GR powder and CBZ powder were both dissolved in distilled water for intragastric administration. The naïve and control groups both received water by intragastric administration. The corresponding treatments lasted for 14 days as pretreatment. On day 14, SE was induced to the following groups: control, GR, and CBZ. In the wake of the SE induction, the mice received with 5 more days of individual treatment.

### 2.4 Status epilepticus and subsequent epilepsy induction

**Figure 1:**
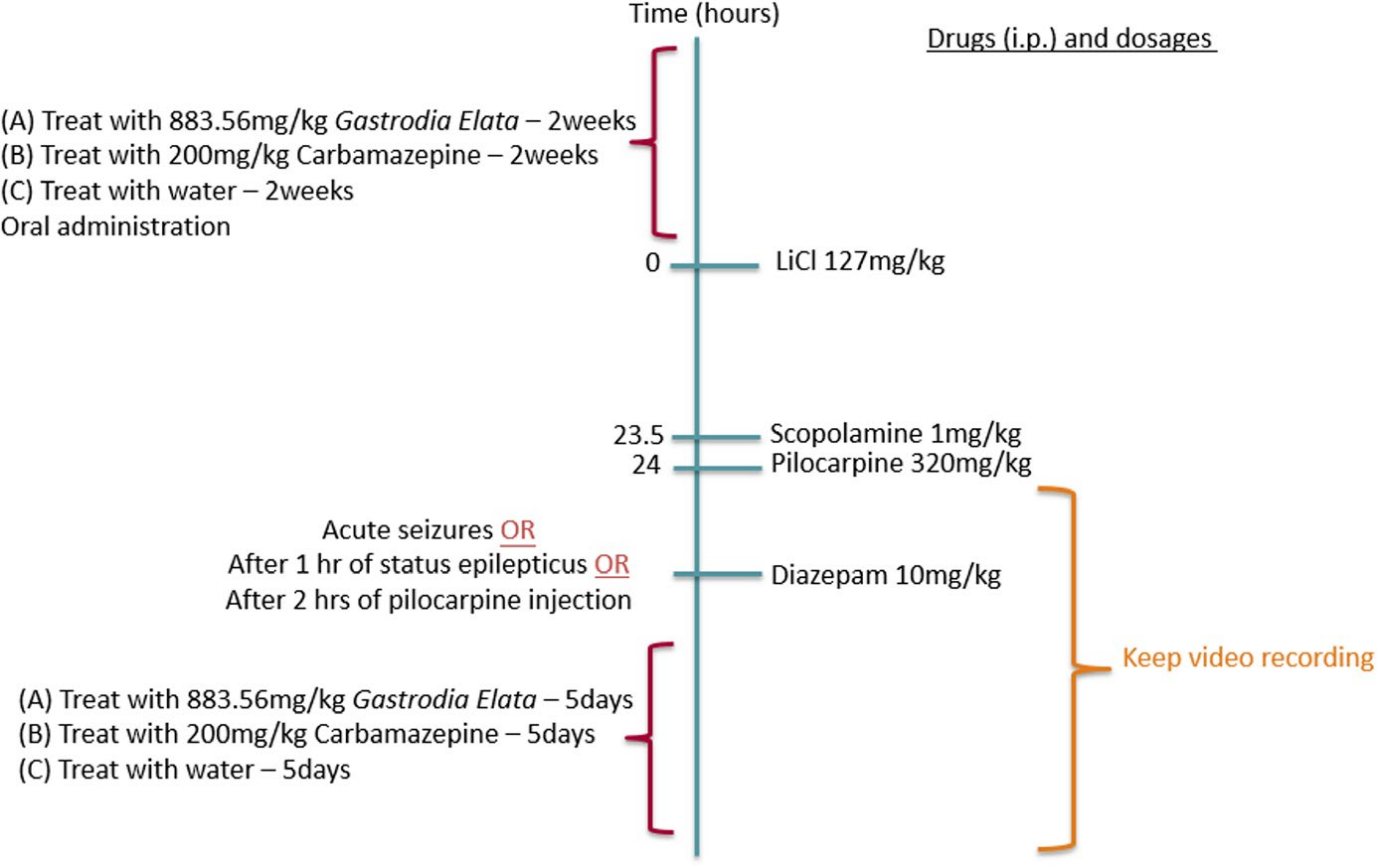
Schematic diagram illustrating the experimental design, treatment schedule, and status epilepticus (SE) induction. Mice were randomly divided into four groups: the control group (n = 12), Gastrodiae Rhizoma group (GR, n = 12), carbamazepine group (CBZ, n = 12), and naïve group (n = 6). Corresponding treatments were given for 14 d prior to SE induction. A single dose of 127 mg/kg lithium chloride (LiCl) (intraperitoneal injection, ip) and a single dose of 1 mg/kg scopolamine (ip) were given in 24 and 0.5 h, respectively, before 320 mg/kg pilocarpine injection with video recording. The occurrence of seizures was scored in accordance with Racine's scale during the induction. Mice received 10 mg/kg diazepam to terminate seizures that were beyond stage 5, 1 h after SE had developed or 2 h after pilocarpine injection. The subsequent treatment period lasted for 5 d after SE induction

Pilocarpine‐induced SE of TLE model was in keeping with the academic community.^14^ Briefly, a single dose of 127 mg/kg lithium chloride (intraperitoneal injection ip, Sigma‐Aldrich) was administered 24 hours before pilocarpine. Mice were then received a single dose of 1 mg/kg methyl scopolamine (ip, Sigma‐Aldrich) 30 minutes before 320 mg/kg pilocarpine (ip, Sigma‐Aldrich) injection to induce SE. Racine's scale was used to classify behavioral seizures after SE induction^16^: Stage 1 consisted of mouth and facial movements. Stage 2 entailed head nodding. Stage 3 may give rise to forelimb clonus. Stage 4 showed rearing. Stage 5 showed both rearing and falling. Stage 4 seizure is considered as the incidence of SE. Seizure episodes were recorded, and the onset time was noted. To conclude the SE induction process, 10 mg/kg diazepam (ip, Sigma‐Aldrich) would be given in one of the following situations: (a) when seizures beyond stage 5 were observed (ie, tonic‐clonic seizures), (b) 1 hour after SE (stage 4) had developed, and (c) 2 hours after pilocarpine injection if no reported incidence of SE. The induction process and the subsequent treatment period were recorded using a camera‐equipped computer for the determination of the occurrence of acute seizures. The induction protocol was illustrated in Figure 1.

### 2.5 Brain tissue sampling and processing

Five days after SE induction, the mice were anesthetized with 80 mg/kg ketamine (ip, Sigma‐Aldrich) and perfused by 40 mL 1x phosphate‐buffered saline (PBS) in body circulation with intracardiac injection. The brains were rapidly removed and were cut into two halves along the sagittal plane. One half was immersed in 4% paraformaldehyde and embedded in paraffin. The embedded half‐brain was then sliced into 6‐µm‐thick sections by microtome sectioning (Shandon™ Finesse™ 325 Manual Microtome, ThermoFisher^TM^) for immunohistochemical study. The other half was snap‐frozen in liquid nitrogen for further protein extraction and enzyme‐linked immunosorbent assay (ELISA).

### 2.6 Whole brain homogenization and protein extraction

Extraction buffer consisted of tissue protein extraction reagents (T‐Per, ThermoFisher^TM^), protease inhibitor cocktail (cOmpleteTM, Sigma‐Aldrich), and phosphatase inhibitor cocktail (PhosSTOPTM, Sigma‐Aldrich). Brain samples were defrosted in ice bath and washed with ice‐cold 1xPBS twice. 500 µL of extraction buffer was added to each sample, which was ground with a polyethylene homogenizer on ice. The brain homogenate was centrifuged at 10 000 *g* at 4°C for 1 hour. The supernatant was collected for protein content measurement.

### 2.7 Measurement of phosphorylated mammalian target of rapamycin (p‐mTOR) and semaphorin 3F (SEMA3F) protein

The expression level of p‐mTOR and SEMA3F protein in brain tissues was determined using mTOR ELISA kit (ab168538, Abcam®) and SEMA3F ELISA kit (LS‐F12580, LSBio) according to manufacturer's instructions. A blank well in each detection was used a control. The absorbance for colorimetric detection was recorded at a wavelength of 450 nm. Bicinchoninic acid protein assay was performed in each group and used to measure the total protein content to normalize the amount of p‐mTOR and SEMA3F.

### 2.8 Immunohistochemistry staining

Brain slides were de‐waxed with xylene for 10 minutes twice. Rehydration of the slides was carried out with the following procedures: 100% ethanol for 5 minutes, 95% ethanol for 5 minutes, 85% ethanol for 5 minutes, and running distilled water for 5 minutes. 1% citrate buffer was used for antigen retrieval by microwave heating for 12 minutes. The slides were washed in 1xPBS and incubated with 0.3% Triton X‐100 for 10 minutes at room temperature to increase the cell permeability. The slides were then blocked by 5% bovine serum albumin (BSA) and 5% normal goat serum for 30 minutes at room temperature and incubated with primary antibodies in a moist chamber at 4°C overnight. The primary antibodies were microtubule‐associated protein 2 (MAP2) (mouse monoclonal, 1:25 dilution, ab11267, Abcam®) and glial fibrillary acidic protein (GFAP) (rabbit polyclonal, 1:50 dilution, ab7260, Abcam®). The primary antibodies were removed and washed with 1xPBS for 5 minutes. The slides were immersed with 0.3% Triton X‐100 for 30 minutes and then washed with 1xPBS for 5 minutes twice. Secondary antibodies containing goat antimouse lgG H&L Alexa Flour® 488 (1:200 dilution, ab150117, Abcam®) and goat anti‐Rabbit lgG H&L Alexa Flour® 594 (1:300 dilution, ab150088, Abcam®) were incubated for 3 hours in a humid atmosphere at room temperature. 1x PBS was used for washing the slides. 0.0001% 4',6‐diamidino‐2‐phenylindole (DAPI, D1306, Invitrogen) was used to counterstain the nuclei for 10 minutes. After the staining, slides were washed with 1xPBS three times and were mounted using Shur/Mount mounting mediawater base (proLab) for perseveration. The samples were observed under a fluorescent microscope (Olympus, IX71) with different filters. Illumination and the size of capturing area were fixed during image acquisition to keep constant of total pixel in each image. Cell quantification was carried out using the Adobe Photoshop software. Red‐Green‐Blue channel in the software was adopted to identify the corresponding pixel area in association with each stained cell types: Green color represented neuronal cells, red color represented reactive astrocytes, and blue color represented cell nuclei. The percentages of positively stained pixel area (red or green or blue) of different mice groups were reported in averaged percentages (calculated from the value of positively stained pixel area divided by the total number of pixels), which were calculated from a collective manner of the same group of mice. All post‐SE survival mice were included for histological analysis (the naïve group n = 6, the control group n = 10, the GR group n = 10, and the CBZ group n = 11). For each mouse, we used one slice for the quantification of the stained pixel area.

### 2.9 Statistical analysis

Data were expressed as mean ± standard deviations (SD). Kaplan‐Meier analysis was used for plotting the time against percentage of mice developing SE and the log‐rank test for comparison among the control group to treatment groups by using Statistical Package for the Social Sciences 23 (SPSS). One‐way analysis of variance (ANOVA) and post hoc Dunnett's test were used to compare among groups in showing their effects on seizure stage development, acute seizure frequency, expression changes in p‐mTOR and SEMA3F, and immunohistochemical studies, using GraphPad Prism 6 software. Outliners were excluded when excessing 95% (mean ± 2SD) interval or preforming as extreme to the dataset. All statistical difference was considered at 5% level of significance.

## 3 RESULTS

### 3.1 Proportion of mice developing SE

**Figure 2:**
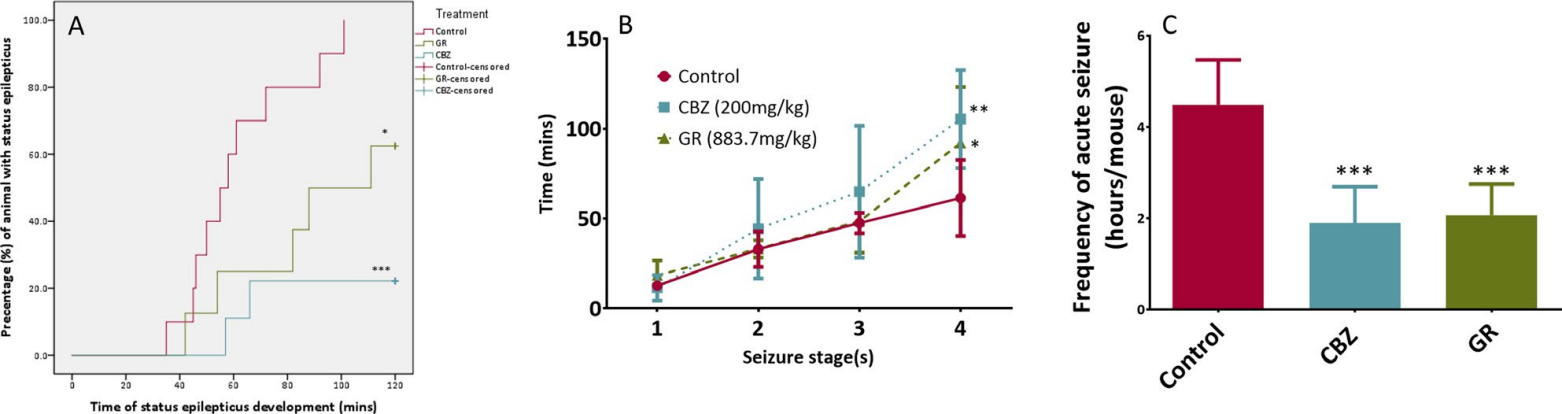
A, Kaplan‐Meier analysis of time to development of status epilepticus (SE). The log‐rank test indicated that mice (n = 12 in each group) administered with carbamazepine (CBZ) and Gastrodiae Rhizoma (GR) had significantly prolonged SE (stage 4 seizure) onset time as compared with the control group, ***P < .001 and *P = .044, respectively. B, Pretreatment effects of Gastrodiae Rhizoma (GR) and carbamazepine (CBZ) on different seizure stages. Data were expressed as mean ± SD and analyzed by one‐way analysis of variance (ANOVA) and post hoc Dunnett's test as compared with the control group (n = 12 in each group). The ANOVA analysis of different groups showed a *P* = .005 in seizure stage 4, indicating that at least one group had a statistically significant difference among others in this stage. In seizure stage 4, a significant difference was found in the comparison between the control group and GR (*P = .041) and CBZ (**P = .004) by post hoc Dunnett's test, indicating a latency to reach status epilepticus progressively. C, Treatment effect of Gastrodiae Rhizoma (GR, n = 11) and carbamazepine (CBZ, n = 10) on attenuating the average frequency of acute seizure; data were expressed as mean ± SD. One‐way analysis of variance (ANOVA) showed a *P* < .001 between all groups, indicating that at least one group had a statistical significance among others. In the post hoc Dunnett's test that compared with the control group (n = 10), a statistically significant difference was found in both the GR and CBZ groups (***P < .001)

In Table 1, the percentage survival was 83.3% (10/12) for both the control and the CBZ groups and 91.6% (11/12) for the GR group after SE induction. The survival rates among the three groups were not significantly different although the GR group had the highest survival rate per se. The percentage of mice developing SE was 83.3% (10/12), 41.7% (5/12), and 17.7% (2/12) for the control, GR, and CBZ groups, respectively. There was a significant reduction in the proportion of mice developing SE for the GR and CBZ groups as compared with the control group. As shown in Figure 2A, the Kaplan‐Meier analysis showed that the percentages of mice developing SE against time in both the CBZ (*P* < .001) and GR groups (*P* = .044) achieved statistical significance using log‐rank test.

**Table 1:** Pilocarpine‐inducted status epilepticus (SE) in altering survival rate and treatment effect of Gastrodiae Rhizoma (GR) and carbamazepine (CBZ) on different seizure stages according to Racine's scale

Groups	Survival rate, n (%)	Latency to first seizure (stage 1) (min ± SD)	Latency to SE (stage 4) (min ± SD)	Percentage of SE induction, n (%)	Seizure frequency h/mouse (number of seizures ± SD)
Naïve (n = 6)	6 (100)	‐	‐	‐	‐
Control (n = 12)	10 (83.3)	12.7 ± 2.4	61.5 ± 21.1	10 (83.3)	4.5 ± 1.0
CBZ (n = 12)	10 (83.3)	11.6 ± 7.1	105.4 ± 27.2**	2 (17.7)	1.9 ± 0.8***
GR (n = 12)	11 (91.6)	18.5 ± 8.3	92.1 ± 31.1*	5 (41.7)	2.1 ± 0.7***

Note

Data were expressed as mean ± SD and analyzed by one‐way analysis of variance (ANOVA) with post hoc Dunnett's test.

Each group contained 12 mice at the beginning of receiving pilocarpine, and the survival rate was 83.3% in the control and CBZ group and 91.6% in GR group.

Significant differences were found in latency to SE in CBZ (n = 12, **P = .004) and GR (n = 12, *P = .041).

No additional mouse was used for compensation of the death; the survived mice were used to carry on the following investigations.

Frequency of acute seizure was decreased with treatment of CBZ (n = 10, ***P < .001) and GR (n = 11, ***P < .001) in compared with the control group (n = 10).

The seizure frequency of each group was counted after SE induction in a fixed period daily (12 noon‐3 pm of the recording time, which was randomly selected in the daytime under the consideration of efficiency to represent the seizure frequency of that day).

### 3.2 Latency of seizure stage development

The overall trend of seizure development was delayed with either GR or CBZ treatment, as shown in Figure 2B. In seizure stage 1, stage 2, and stage 3, the latency was not significant for all groups. The latency to reach stage 4 seizure in the CBZ group (105.4 ± 27.2 minutes, *P = *.004) was longer than that of in the control group (61.5 ± 21.1 minutes). Statistical significance of latency to reach stage 4 seizure was achieved in the GR group (92.1 ± 31.1 minutes, *P* = .041) in comparison with the control group (Table 1).

### 3.3 Suppression of acute seizures

The acute seizures were noted in all post‐SE survival mice. The antiepileptic effect was evaluated based on counting of seizures in a fixed 3 hours of period daily (12 noon‐3 pm of the recording time, which was randomly selected in the daytime under the consideration of efficiency to represent the seizure frequency of each day), and 5 days of recording was counted after SE. Figure 2C showed that both the GR and CBZ groups had a lower seizure frequency than the control group. The seizure frequency was 4.5 ± 1.0, 2.1 ± 0.7, and 1.9 ± 0.8 (h/mouse) in the control, GR, and CBZ groups, respectively (Table 1). For both GR and CBZ treatments, a significant seizure reduction (*P* < .001) was observed as compared with the control group as shown in Figure 2C.

### 3.4 Downregulation of p‐mTOR

**Figure 3:**
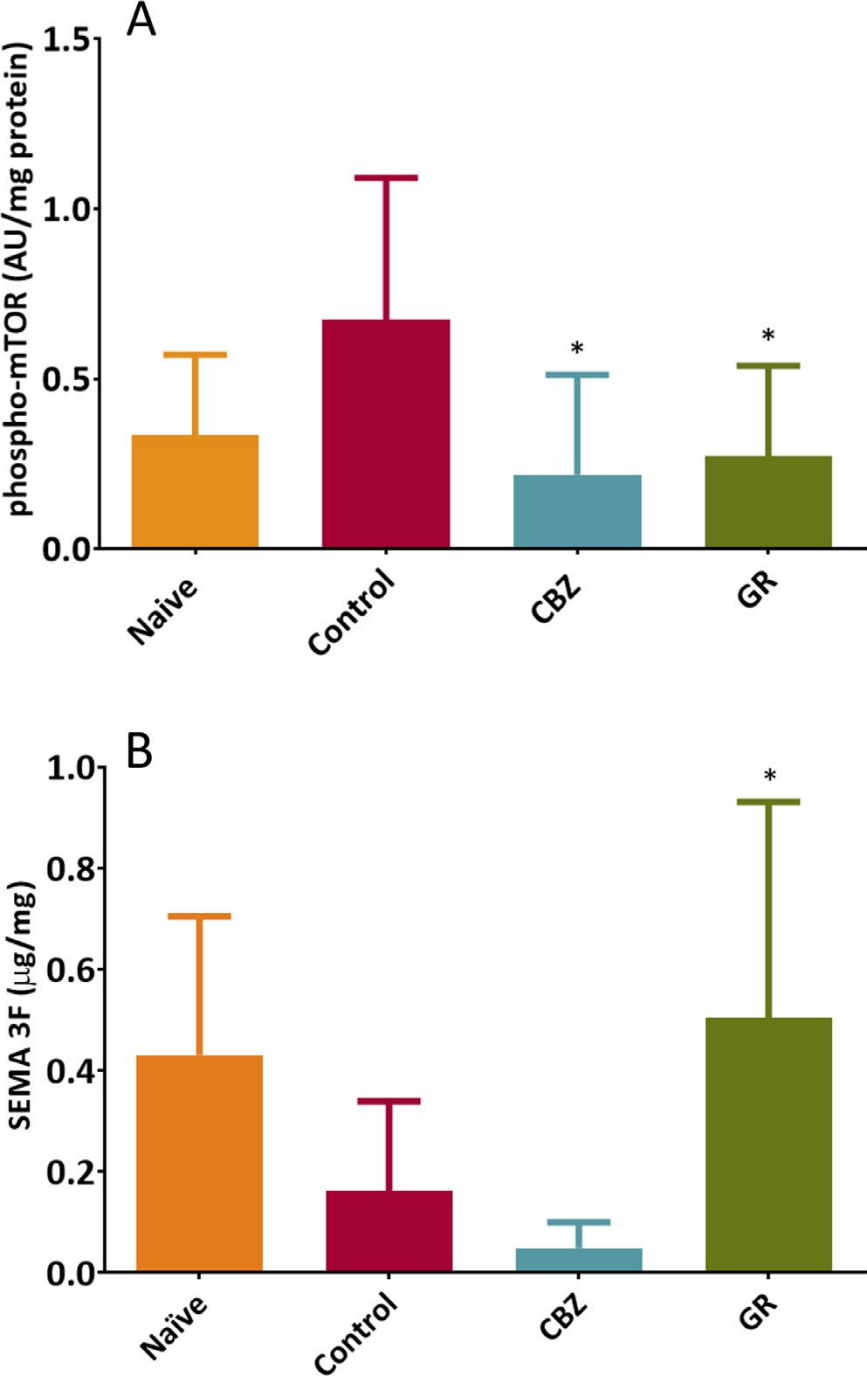
A, The regulatory effect of Gastrodiae Rhizoma (GR) and carbamazepine (CBZ) on p‐mTOR expression. Data were shown as mean ± SD; negative absorbance was not excluded from the analysis. The naïve group (n = 6) represented the normal expression. The control group represented the expression in an epileptic condition. One‐way analysis of variance (ANOVA) showed a *P = *.048 between all groups, indicating that at least one group had a statistical significance among others. Treatment groups were compared with the control group (n = 8; two outliers were excluded) by post hoc Dunnett's test. The treatment effect of GR (n = 9; one outlier was excluded, *P = .047) and CBZ (n = 6; one outlier was excluded, *P = .041) could significantly suppress the expression of p‐mTOR. B, The regulatory effect of Gastrodiae Rhizoma (GR) and carbamazepine (CBZ) on semaphorin 3F (SEMA3F) expression. Data were reported as mean ± SD. The naïve group (n = 6) represented the normal expression, while the control group (n = 8, two outliers were excluded) represented the expression in an epileptic condition. One‐way analysis of variance (ANOVA) showed a *P = *.005 between all groups, indicating that at least one group had a statistical difference among others. The treatment effect of GR and CBZ was compared with the control group by post hoc Dunnett's test. GR treatment would upregulate the expression of SEMA3F (n = 10, one outliner was excluded, *P = .034). On the other hand, the treatment with CBZ trend to reduce the expression of SEMA3F without statistics significant (n = 10, *P* = .716)

The baseline level of p‐mTOR was 0.33 ± 0.24 AU/mg protein in the naïve group that represented a normal expression level of p‐mTOR. Status epilepticus induction upregulated the level of p‐mTOR to 0.67 ± 0.42 AU/mg protein in the control group. This pathological change in aberrant p‐mTOR level was found to be downregulated by the treatment of GR or CBZ significantly. The level of p‐mTOR was 0.27 ± 0.27 AU/mg protein in GR treatment group (*P* = .047) and 0.22 ± 0.29 AU/mg protein in CBZ treatment group (*P* = .041) as compared with the control group (Figure 3A).

### 3.5 Upregulation of SEMA3F

The quantification of the SEMA3F level was shown in Figure 3B. The baseline expression of SEMA3F was 0.43 ± 0.27 µg/mg protein in the naïve group. SE induction downregulated the level of SEMA3F in the control group (0.16 ± 0.18 µg/mg protein). Treatment of GR upregulated the underexpression of SEMA3F significantly to 0.51 ± 0.43 µg/mg protein (*P* = .034) as compared with the control group. CBZ decreased the level of SEMA3F as compared with the control group. This result was not significant (0.05 ± 0.05 µg/mg, *P* = .716).

### 3.6 Histological changes in hippocampus: neuronal loss and astrogliosis

**Figure 4:**
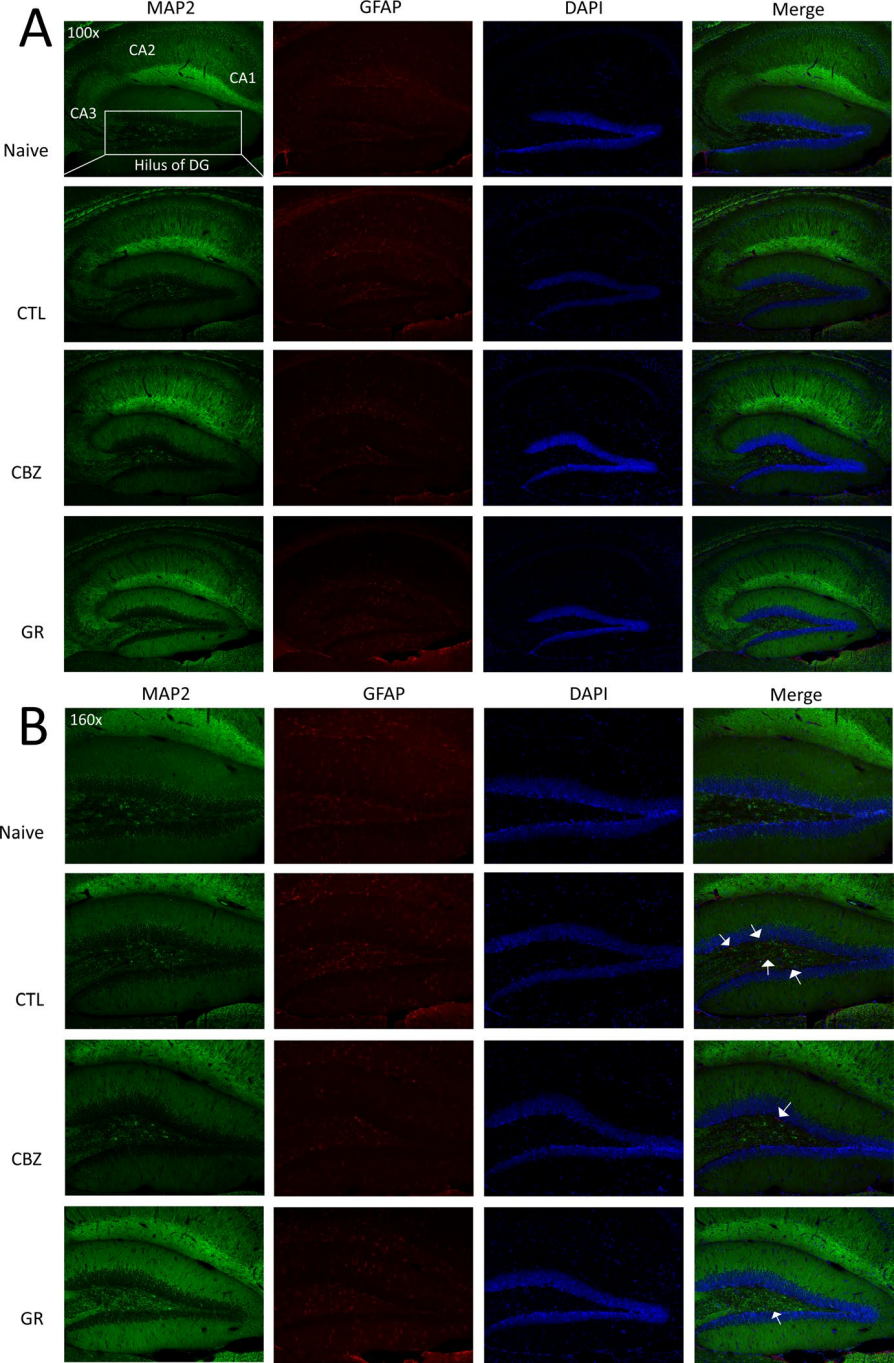
Representative images of (A) hippocampal (100x) and (B) hilus of dentate gyrus (160x) histopathology 5 days after the status epilepticus induction. The brain specimens were cut in a longitudinal plane in 6 µm thickness. The quantification of the pixel area in neuronal cells (microtubule‐associated protein 2, MAP2 positive) was shown in green color; astrocytes (glial fibrillary acidic protein, GFAP positive) were shown in red color, and the nuclei (4',6‐diamidino‐2‐phenylindole, DAPI positive) was shown in blue color. One slice from each mouse was used for quantification of the stained pixel area. The naïve group represented the histochemistry in normal condition, while the control group represented an epileptic condition. Alleviation of active astrocytes was showed by treating with Gastrodiae Rhizoma (GR) and carbamazepine (CBZ); arrows point to indicate the active astrocytes in the hilus of the dentate gyrus

**Figure 5:**
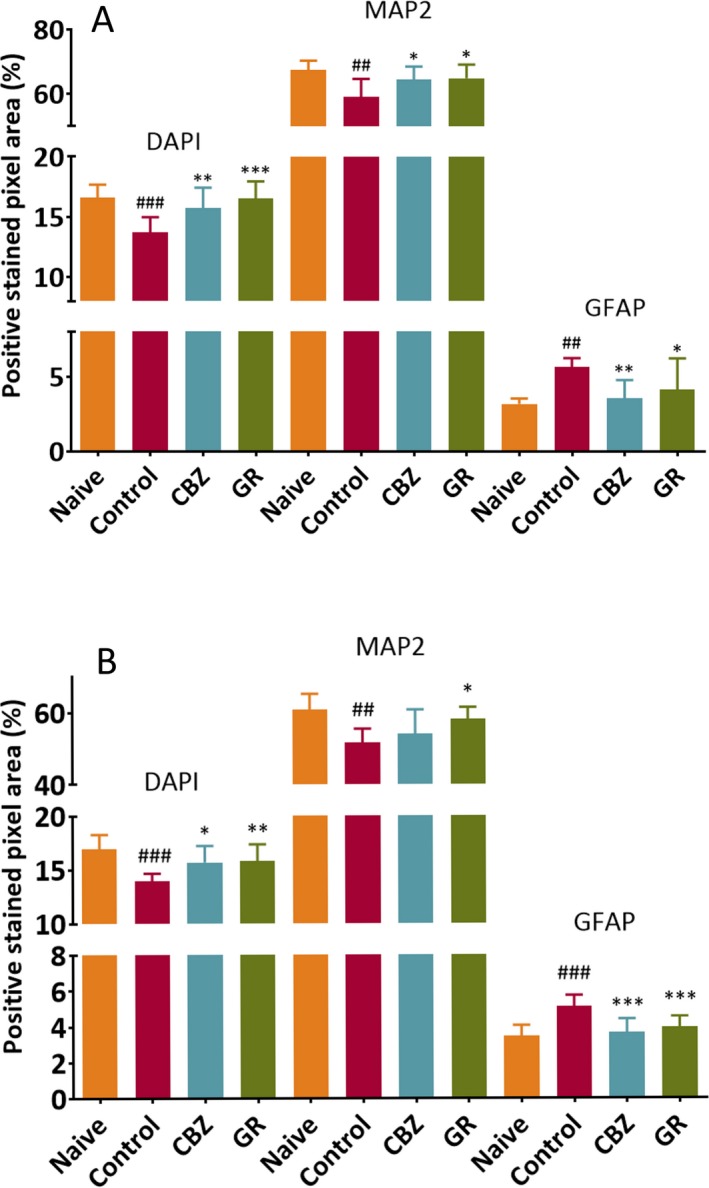
A, Effect of Gastrodiae Rhizoma (GR) and carbamazepine (CBZ) on neuron reservation and astrogliosis alleviation in the hippocampus. Immunostaining of neurons (microtubule‐associated protein 2, MAP2 positive), reactive astrocytes (glial fibrillary acidic protein, GFAP positive), and nuclei of cells (4', 6‐diamidino‐2‐phenylindole, DAPI positive) were quantified in area of interested. Data were expressed as means ± SD and analyzed by one‐way analysis of variance (ANOVA) and post hoc Dunnett's test. One slice from each mouse was used for quantification of the stained pixel area. The naïve group (n = 6) represented the cell distribution in normal condition, while the control group (n = 10) represented an epileptic condition, which had a significant decrease in neurons (^##^ *P* = .002) and a significant increase in active astrogliosis (^##^ *P* = .004) as compared with the naïve group. In the GR (n = 10) and CBZ (n = 11) groups, both reserved neurons (*P = .014, *P = .028, respectively) and alleviated astrogliosis (*P = .047, **P = .004, respectively) as compared with the control group. B, Effect of Gastrodiae Rhizoma (GR) and carbamazepine (CBZ) on neuron reservation and astrogliosis alleviation in the hilus of dentate gyrus. Immunostaining of neurons (microtubule‐associated protein 2, MAP2 positive), reactive astrocytes (glial fibrillary acidic protein, GFAP positive), and nuclei of cells (4', 6‐diamidino‐2‐phenylindole, DAPI positive) were quantified in area of interested. Data were expressed as means ± SD and analyzed by one‐way analysis of variance (ANOVA) and post hoc Dunnett's test. One slice from each mouse was used for quantification of the stained pixel area. The naïve group (n = 6) represented the cell distribution in normal condition, while the control group (n = 10) represented an epileptic condition, which had a significant decrease in neurons (^##^ *P* = .002) and a significant increase in active astrogliosis (^###^ *P* < .001) as compared with the naïve group. In the GR (n = 10) and CBZ (n = 11) groups, they both alleviated astrogliosis (***P ≤ .001) and only GR could reserve neuronal density (*P = .012) as compared with the control group

The stained pixel areas of MAP2 and GFAP represented neurons and reactive astrocytes, respectively. The stained pixel area of interest was quantified and reported as an averaged percentage. The most representing image from each group was selected and shown in Figure 4A. In the naïve group (which did not receive SE induction or subsequent treatment), the proportion of MAP2 stained pixel area outweighed that of GFAP‐stained pixel area. In the control group (which received SE induction), we found histological changes after SE induction. The averaged pixel areas of MAP2‐positive cells were significantly lower in the control group (59.0 ± 5.7%) when compared with that of the naïve group (67.5 ± 2.8%, *P* = .002). The averaged pixel areas of GFAP‐positive cells were significantly higher in the control group (5.6 ± 0.6%) when compared with that of the naïve group (3.2 ± 0.4%, *P* = .004). These results were in consonance with pathological features of TLE patients. The effects of CBZ and GR on the pathological changes were compared with the control group and shown in Figure 5A. The stained pixel area of MAP2 was significantly increased by both CBZ (64.4 ± 4.1%, *P* = .028) and GR (64.8 ± 4.2%, *P* = .014). On the other hand, the stained pixel area of GFAP was mitigated in both CBZ (3.5 ± 1.2%, *P* = .004) and GR (4.1 ± 2.1%, *P* = .047).

### 3.7 Histological changes in the hilus of dentate gyrus: neuronal loss and astrogliosis

By comparing the naïve group with the control group, similar pathological changes were observed in the hilus of dentate gyrus (DG) (Figure 4B): SE decreased the percentage of MAP2 stained pixel area (51.6 ± 3.8%, *P* = .002) and increased the percentage of GFAP‐stained pixel area (5.1 ± 0.6%, *P* < .001) in the control group (Figure 5B). The neuronal loss was significantly ameliorated by GR (58.0 ± 3.4%, *P* = .012) as compared with the control group, whereas CBZ treatment could not prevent the neuronal loss (53.9 ± 6.9%, *P* = .581). A statistically significant difference was found in the reduction of the percentage of GFAP‐stained pixel area in both the CBZ (3.7 ± 0.8%, *P* < .001) and GR (4.0 ± 0.6%, *P* = .001) groups as compared with the control group.

## 4 DISCUSSION

Our results revealed that GR treatment could prevent a large proportion of mice from developing seizure at different stages of Racine's scale for pilocarpine‐induced TLE. Moreover, the onset time of SE could be delayed. In the post‐SE stage, acute seizures were attenuated. It implies that GR has a potential role in a TLE model of seizures. GR may downregulate the overexpressed levels of p‐mTOR. This downregulation of p‐mTOR also appears to reduce the severity of seizures. GR may upregulate the underexpression levels of SEMA3F. There could be a potential association with mossy fiber modulation. The regulation of p‐mTOR and SEMA3F may accompany by the antiepileptic effect of GR. Our histological data further suggested that GR may prevent neuronal loss and mitigate astrogliosis in the hippocampus. These effects are similar in the hilar region of the hippocampus. With the support of all findings, we suggested that GR has antiepileptic properties which could be useful for translation into clinical use.

We adopted the pilocarpine‐induced SE model of mice, which is a well‐established chemical‐induced model for TLE. Although the use of lithium is not universal, recent studies used lithium, combined with scopolamine, to reduce the overall pilocarpine dose in a mice TLE model.^17^ Pilocarpine is a muscarinic acetylcholine receptor agonist acting on M1 subunit of G protein–coupled receptors that damages the hippocampus, resulting in the generation of SE.^18^, ^19^ The model resembles clinical features by producing an injury that leads to an acute phase with symptomatic seizure, followed by recurrent seizures in the chronic phase.^20^ In particular, a bi‐phasic appearance was found for spontaneous recurrent seizures after SE induction: An early peak was found on days 1‐3 after SE induction, and a later peak was found on days 15‐20 after SE induction.^21^ Pilocarpine‐induced SE is associated with neuronal injury, and there may be proliferation reactive astrocytes and mossy fiber sprouting in the area of Cornu Ammonis and the hilus of DG^22^, ^23^ that are hallmarks of pathophysiological change in TLE and hippocampal sclerosis. The use of this model in testing the antiepileptic effects of GR is appropriate, and it contributes toward our understanding of TLE.

The degree of lesion in the hippocampus is associated with neuronal loss and astrogliosis that correlated with seizure severity. that astrocytic dysfunction would strengthen the release of glutamate and intensify Wetherington et al demonstrated neuronal excitability, thus leading to seizures.^24^, ^25^, ^26^ Our results illustrated that mice with fewer seizures had less severe neuronal atrophy and astrogliosis in their hippocampus and the hilus of DG, implying that GR could modulate SE formation and contribute to seizure maintenance. Our findings were consistent with Kim et al,^8^ who described that methanol extract of GR protected neurons from excessive neuronal excitation. They reported that the protective effects of GR were related to the reduction of the seizure frequency.^8^ Han discovered similar protective effects of GR—the methanol extract reduced glutamate‐induced oxidative toxicity within HT22 cellular network.^27^ Moreover, the correlation of mitigating proliferation of reactive astrocytes and seizure frequency was pointed out by Heja et al,^28^ who proved that inhibiting the growth of reactive astrocytes caused a reduction of recurrent seizures. When compared with the above studies, our research showed feasibility in the use of water extract of GR in a pilocarpine TLE mice model. The water extract of GR is easier to extract in noncommercial industries, and it has less absorption and safety concerns. With the evidence of our findings and other studies in the literature, a noticeable interest was that suppressing neuronal loss and astrogliosis physiologically would accomplish seizure reduction. Our findings implied a potential role of GR in preventing neuronal atrophy, astrogliosis, and possible subsequent attenuation of seizure frequency and development of hippocampal sclerosis. These observations were also shared by other research groups who have found a considerable number of therapeutic targets for acquired epilepsies.^26^

mTOR maintains neuronal balance and regulates synaptic excitability in the brain. Reactive astrocyte could activate the pathway of p‐mTOR excessively, leading to seizure.^29^ It was previously postulated that rapamycin could suppress astrogliosis and seizures through the inhibition of p‐mTOR.^30^ However, only a few studies reported the mechanisms which were closely associated with mTOR pathway. Our results showed a reduction in p‐mTOR level after GR treatment. We verified that GR was a potential inhibitor which could reduce the excessive levels of p‐mTOR. Other studies were pivotal in our hypothesis that GR could regulate p‐mTOR level by multiple signaling mechanisms, including PI3K and MAPK.^10^, ^11^, ^30^. Hsieh et al^10^ discovered that the aqueous extract of GR regulates AP‐1 expression via c‐Jun N‐terminal kinase (JNK) and the p38 pathway to overcome excitation of glutamate in kainic acid‐induced epileptic rats. Jiang et al^31^ found that gastrodin inhibits glutamate‐induced p38 and MAPK activation toward cell death. The mTOR and MAPK pathways are associated with cellular signaling (ie, oxidative stress and growth factors) which guided RNA‐binding proteins, Hu family, and fragile X mental retardation protein (FMRP), to regulate the synaptic formation causing epileptic excitability.^32^, ^33^, ^34^ In particular, the MAPK mechanism consists of three downstream pathways: the JNK, the p38, and the extracellular signal‐regulated kinase (ERK) pathways. In local gene expression on synapses, the ERK pathway is involved with the synthesis of *N*‐methyl‐D‐aspartic acid (NMDA) receptor that may contribute toward synaptic excitability and the expression level of mTOR maintained the synthesis of GABAergic and glutamatergic signaling, both of which may contribute toward epilepsy formation.^35^, ^36^ The seizure outcomes of the mice with GR treatment were accompanied by a reduction of the p‐mTOR level. Citraro et al^37^ reviewed that excessive activation of p‐mTOR enhanced seizure frequency and p‐mTOR inhibitors could resolve the symptoms. Inhibition of p‐mTOR may involve in suppressing of aberrant positive feedback circuits in mossy fiber sprouting and seizures.^38^, ^39^ Thus, it is reasonable to consider that the antiepileptic effect of GR may relate to modulating overexpression p‐mTOR to achieve seizure control and astrogliosis.

The phenomenon of axonal sprouting compensates for neuronal loss in the hippocampus, which worsens the seizures in TLE.^40^, ^41^ This phenomenon has been reported in pilocarpine‐induced SE models previously in the response of astrogliosis.^42^, ^43^ The expression of class III semaphorin protein family guides axonal growth and synaptic formation, resulting in downstream signaling components which regulate synaptic physiology and neuronal excitability.^44^, ^45^ In particular, literature illustrated that SE facilitated the synaptic reorganization of epileptic circuitry and a decrease in SEMA3F level, resulting in axonal sprouting toward mossy fiber sprouting.^46^, ^47^ Fu et al documented that SEMA3F mediated downscaling α‐amino‐3‐hydroxy‐5‐methyl‐4‐isoxazolepropionic acid (AMPA) receptor as feedback mechanism to regulate synaptic activity.^48^ To understand the roles of GR on axonal sprouting, we found that GR upregulated SEMA3F. These results showed that GR may mitigate the downregulated SEMA3F and could prevent the abnormal growth of axons. Our histological findings discovered a similar observation on the preservation of neurons. It showed that GR could reserve the neurons against pilocarpine‐induced SE and would further reduce the progression of axonal sprouting via a regulatory role on SEMA3F. However, the possible upstream pathway on regulating SEMA3F and progression of axonal sprouting needs further experiments to conclude.

Our data illustrated that GR delayed the development of SE and suppressed acute seizures, showing the antiepileptic effects. GR downregulated the overexpression of p‐mTOR level and upregulated the underexpression of SEMA3F level. These results may attribute to the antiepileptic effects by producing less initial insult. GR could relieve the pathological changes in TLE. Altogether, we concluded that the antiepileptic effect of GR could accompany the mTOR reduction and astrogliosis attenuation. In general, AEDs would not be prescribed for the sole purpose of preventing epileptogenesis.^49^ Our study has found considerable uses of GR as an option beyond AEDs in terms of treatment of TLE. We recommend a systematic pharmacological profiling study for GR, whereby the safe dose, adverse effects, and herb‐drug interactions of GR with AEDs could be determined. An extended observation to conclude the effect of GR in chronic seizure remains essential tasks for the future.

## CONFLICT OF INTERESTS

The authors declared that there is no conflict of interest. We confirm that we have read the Journal's position on issues involved in ethical publication and affirm that this report is consistent with those guidelines.
